# Image-guided percutaneous ablation for lung malignancies

**DOI:** 10.3389/fonc.2022.1020296

**Published:** 2022-11-10

**Authors:** Youlan Shang, Ge Li, Bin Zhang, Yuzhi Wu, Yanjing Chen, Chang Li, Wei Zhao, Jun Liu

**Affiliations:** ^1^ Second Xiangya Hospital, Central South University, Changsha, China; ^2^ Xiangya Hospital, Central South University, Changsha, China

**Keywords:** lung malignancies, lung tumor ablation, radiofrequency ablation, microwave ablation, cryoablation, combination therapy

## Abstract

Image-guided percutaneous lung ablation has proven to be an alternative and effective strategy in the treatment of lung cancer and other lung malignancies. Radiofrequency ablation, microwave ablation, and cryoablation are widely used ablation modalities in clinical practice that can be performed along or combined with other treatment modalities. In this context, this article will review the application of different ablation strategies in lung malignancies.

## Introduction

Primary lung cancer is the leading cause of cancer-related death in both males and females, posing a severe threat to human health (1). In addition, the lung is one of the most frequent sites of metastasis for malignancies. Regarding metastatic lung malignancies, breast, colorectal, prostate, kidney, and bladder cancers, as well as sarcomas, are common primary malignancies. Although surgical resection is of great significance in the treatment of primary and metastatic lung cancer, many patients do not have the opportunity to undergo surgery due to advanced age, comorbidities, poor cardiopulmonary function, or refusal to undergo surgery (2). In addition, chemotherapy and radiotherapy are commonly used to treat lung malignancies, and stereotactic body radiotherapy is an effective treatment for inoperable early-stage NSCLC, but these treatments have their limitations (3). Consequently, it is crucial to identify novel therapeutic approaches to improve the survival of patients with unresectable pulmonary malignancies.

**Figure 1:**
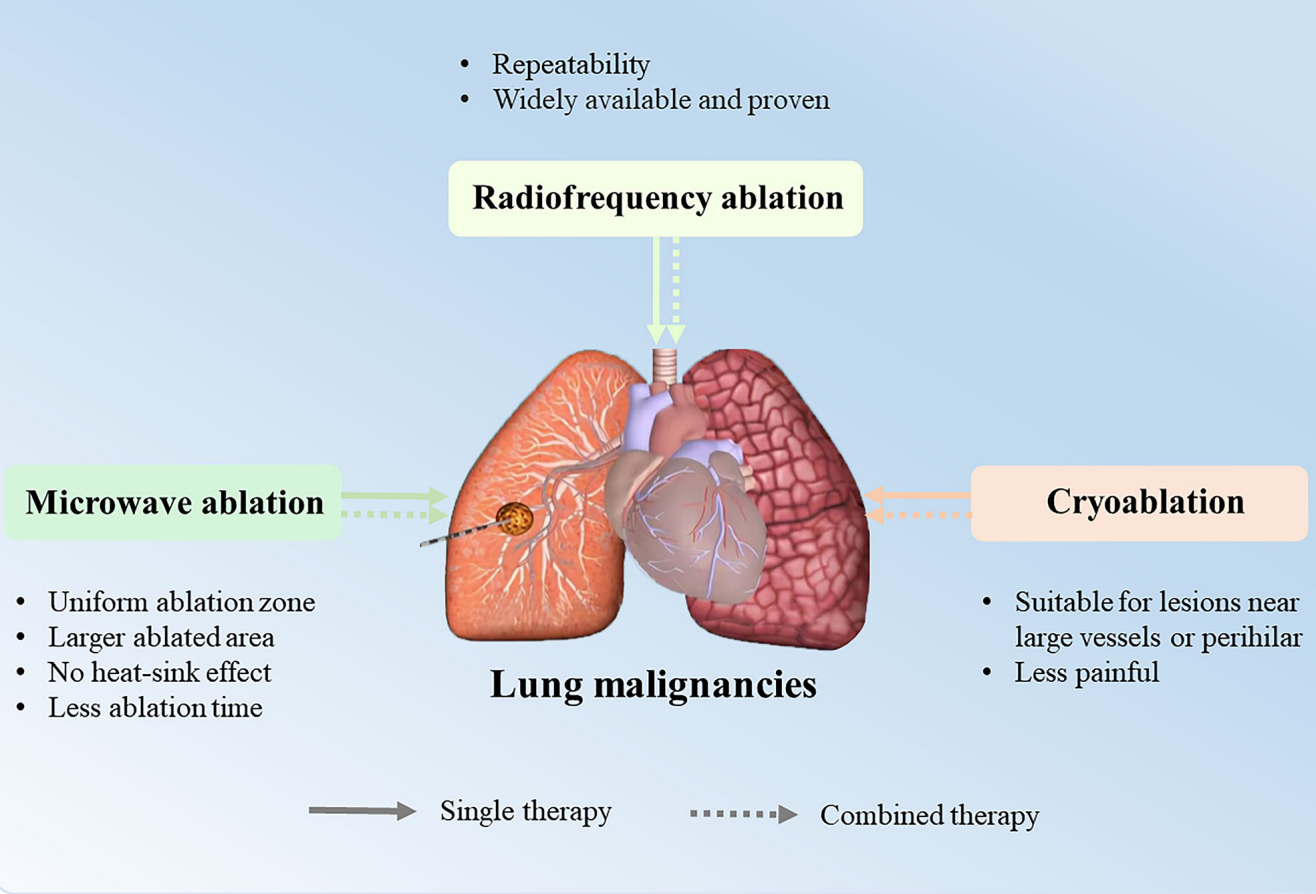
A graphical representation of the summary of the technology. Three ablation modalities: radiofrequency ablation, microwave ablation and cryoablation alone or in combination with other treatment modalities for lung malignancies, and their respective advantages.

In recent years, image-guided percutaneous lung ablation has made great progress and is becoming a very promising treatment regimen. As a minimally invasive treatment, its feasibility and safety have been demonstrated in the treatment of small-sized lung tumors, especially those < 3 cm in diameter. Radiofrequency ablation (RFA), microwave ablation (MWA), and cryoablation (CA) are the three most widely used ablation modalities in the lung, all of which are thermal ablation modalities that destroy tumor cells by applying extreme temperatures directly to the tumor and safety margin (4, 5). Lung ablation treatment can be used as an alternative treatment for patients with inoperable lung cancer in stages I to II to improve disease-free survival and as an adjuvant treatment for patients with advanced stage III to IV lung cancer to alleviate tumor-related symptoms (6). However, certain challenges remain regarding the use of ablation as a single cancer treatment, as it may eventually lead to tumor recurrence due to incomplete ablation around the tumor. Therefore, development of a novel treatment protocol that combines ablation with other therapies is one of the current cancer treatment priorities (7). **Figure 1** shows a graphical representation of the summary of the technology discussed in the review.

## Procedure

Before tumor ablation, patients are evaluated in a clinical setting and undergo a medical history, physical examination, laboratory tests, pathology, and preoperative imaging. It is necessary to inform the patient and/or his/her family about the risks and benefits of surgery, explain the possible complications, and have them sign an informed consent form. Patients are required to fast 4 hours before local anesthesia or 12 hours before general anesthesia to reduce the possibility of nausea or inhalation of stomach contents caused by sedatives.

Computed tomography (CT) is the most common and accurate image-guided technique in lung tumor ablation. Regardless of the ablation modality, imaging is used to guide the placement of one or more applicators into the target tumor or adjacent structures. The patient was placed on the CT scanning table on the day of surgery. General or local anesthesia was selected for surgery according to the patient’s condition. Before ablation, the corresponding preoperative plan was determined, as follows: (i) the location, size, and shape of the tumor and its relationship with adjacent tissues were determined through CT scanning; (ii) the proper body position and puncture site on the body surface were determined; and (iii) the puncture path and ablation parameters were identified. The path should be the shortest possible and avoid important structures. At the time of ablation, according to the preoperative plan, the applicator is used to puncture the target tumor layer by layer along the puncture path, and 3D reconstruction images are used to observe whether the applicator is punctured into the target tumor. Then, the target tissue is ablated according to the size and location of the tumor. Intraoperative CT is used to monitor the extent of ablation and the occurrence of complications, such as bleeding and pneumothorax.

At the end of the surgery, large-scale CT scans are repeated to assess the immediate response through (i) an initial assessment of technical success; (ii) observation of residual ablation; and (iii) identification of any complications. Patients with normal blood pressure, heart rate, oxygen saturation, and no hemoptysis, shortness of breath, chest tightness, or dyspnea can return to the ward. Contrast-enhanced chest CT is the current standard method to evaluate the efficacy of the technique. It is performed monthly for the first three months postoperatively. Subsequently, enhanced chest CT or PET/CT scans and tumor markers are examined every three months to detect whether the local lesions have been completely ablated or whether new pulmonary lesions or extrapulmonary metastases have appeared (8–12).

## Radiofrequency ablation (RFA)

**Figure 2:**
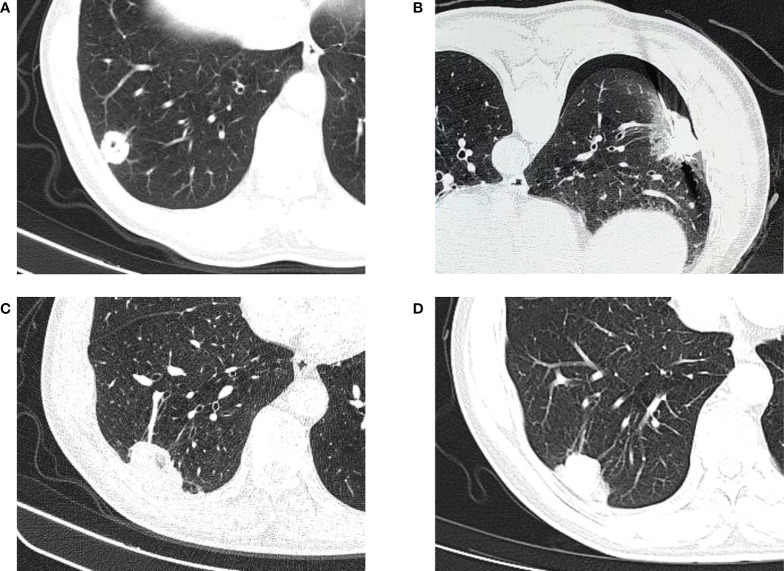
CT-guided percutaneous RFA. **(A)** CT scan performed before RFA, showing a lesion in the lower right lobe. **(B)** CT−guided percutaneous RFA of the lesion. **(C)** CT scan performed 1 month after the procedure. **(D)** CT scan performed 4 months after the procedure.

RFA is the first thermal ablation applied to the lungs. Since Dupuy et al. reported the first three cases of lung cancer treated with RFA in 2000, RFA has been increasingly used in patients with primary and metastatic lung cancer who are not candidates for surgical resection (13). It works by inserting the RF electrode into the tumor tissue and applying an alternating current to generate an electric field oscillating between 375 and 500 MHz, which causes molecules in the tumor tissue to rub and collide with each other to produce heat; when the local temperature reaches 60 °C, coagulative necrosis will occur in the tumor tissue (10, 14). However, the heat dissipation effects of neighboring blood vessels or airways will reduce this thermal energy. Numerous studies have evaluated the safety and efficacy of RFA, as well as assessed its benefits. The main advantage of RFA is experience, as numerous studies have been conducted to evaluate the safety and efficacy of this treatment (15). The RFA process is displayed in **Figure 2**. Several studies of RFA in lung tumors are summarized in **Table 1**.

**Table 1:** Summary of published studies of RFA in lung tumors.

Author, Year	Type	Therapy	Tumor	No. of Patients	Median/mean follow-up (mo)	Mean size (cm)	Median OS (mo)	OS (%)			
								1- year	2-year	3-year	5- year
Simon et al., 2007 (16)	R	RFA	Stage I NSCLC	75	20.5	3.0	29.0	78.0	57.0	36.0	27.0
Lencioni et al., 2008 (17)	P	RFA	NSCLC	33	15.0	2.5	–	70.0	48.0	–	–
Dupuy et al., 2015 (18)	P	RFA	Stage I NSCLC	51	24.0	2.0	–	86.3	69.8	–	–
Gobara et al., 2016 (19)	P	RFA	Stage I NSCLC	33	37.0	1.5	–	97.0	82.0	74.0	–
Huang et al., 2018 (20)	R	RFA	Stage I NSCLC	50	46.9	2.2	47.0	96.0	86.5	67.1	36.3
Dupuy et al., 2006 (21)	P	RFA & radiotherapy	Stage I NSCLC	24	26.7	3.4	–	–	50.0	–	39.0
Chan et al., 2011 (22)	R	RFA & radiotherapy	Stage I NSCLC	17	22.0	3.0	21.0	–	–	–	–
Steber et al., 2021 (23)	P	RFA & EBRT	Early-stage NSCLC	12	–	–	53.6	–	–	–	–
de Baère T et al., 2015 (24)	R	RFA	Metastatic lung tumors	566	35.5	1.5	62.0	92.4	79.4	67.7	51.5
Lencioni et al., 2008 (17)	P	RFA	Metastatic lung tumors	53	15.0	1.3	–	89.0	66.0	–	–
Matsui et al., 2015 (25)	R	RFA	Metastatic lung tumors	84	37.5	1.2	67.0	95.2	–	65.0	51.6
Hiyoshi et al., 2019 (26)	R	RFA	Metastatic lung tumors	43	24.3	1.2	52.7	–	–	–	–
Zhong et al., 2020 (27)	R	RFA	Metastatic lung tumors	60	45.5	1.4	52.0	96.7	–	74.7	44.1
Chua et al., 2010 (28)	R	RFA & chemotherapy	Metastatic lung tumors	100	23.0	–	36.0	87.0	66.0	50.0	30.0
Hasegawa et al., 2021 (29)	R	RFA& surgery	Metastatic lung tumors	17	34.0	–	–	100.0	–	88.0	88.0

RFA, radiofrequency ablation; NSCLC, non-small cell lung cancer; EBRT, external beam radiation therapy; mo, months; OS, overall survival; P, prospective; R, retrospective.

### RFA in early-stage lung cancer

Several studies have demonstrated the safety and efficacy of RFA in the treatment of early-stage non-small cell lung cancer (NSCLC). According to the first published retrospective study, the 1-, 2-, and 3-year overall survival (OS) rates after RFA of early NSCLC were 78%, 57% and 36%, respectively, and the local recurrence rates were 12%, 18%, and 21%, respectively (16). An early prospective multicenter trial, the Rapture study, published in 2008, indicated that NSCLC patients treated with RFA had a 1-year OS of 70% and a 2-year OS of 48%, with stage I NSCLC patients having a 2-year OS and cancer-specific survival rate of 75% and 92%, respectively (17). Several subsequent studies have reported comparable results, showing that RFA can improve the OS of early-stage NSCLC and can reduce the risk of local recurrence. Dupuy et al. reported that the 1- and 2-year OS rates were 86.3% and 69.8%, respectively, and local tumor recurrence-free rates were 68.9% and 59.8%, respectively, among which the OS of tumors with a maximum diameter of < 2 was as high as 83% (18). A prospective multicenter study by Gobara et al. reported OS rates of 97%, 82%, and 74% at 1, 2, and 3 years after RFA in 33 patients with stage IA NSCLC (19). Similarly, Huang et al. discovered that the OS rates at 1, 2, 3, and 5 years after RFA of patients with stage IA NSCLC were 96.0%, 86.5%, 67.1%, and 36.3%, respectively, while the progression-free survival (PFS) rates were 94.0%, 77.5%, 43.5%, and 10.8%, respectively (20). These studies indicate that the maximum tumor diameter is the most critical factor in predicting technical and therapeutic success. According to the American College of Chest Physicians, tumors < 3 cm in diameter appear to be more susceptible to successful treatment (30, 31). In addition, OS has increased gradually between early published research and recent studies. This increase may be due to the gradual improvement of ablation technology and patient selection.

Combining RFA with other treatment modalities for some patients with early-stage lung cancer is viable. Dupuy et al. were the first to report the feasibility of RFA in combination with conventional radiotherapy for inoperable stage I NSCLC, demonstrating better local control and survival than radiotherapy alone (21). Another study also confirmed the efficacy of RFA combined with high-dose rate brachytherapy in the treatment of early-stage NSCLC, with a 53% actuarial survival rate and a 21-month median OS in 17 patients treated with RFA and high-dose rate brachytherapy (22). Steber et al. explored the combination of external beam irradiation therapy (EBRT) and RFA for the treatment of early-stage NSCLC. They reported a median OS of 53.6 months and a median PFS of 11.3 months, indicating that the combination of RFA and EBRT appears to be feasible, with reasonable long-term local control. However, this combination is not recommended because SBRT alone has comparable or superior control rates (23). Moreover, a recent study demonstrated that RFA combined with radioactive particle implantation is superior to RFA alone for the treatment of NSCLC, allowing for improved local short-term outcomes (32).

### RFA in advanced lung cancer

The standard treatment for unresectable advanced NSCLC includes radiotherapy, chemotherapy, molecular targeted therapy (such as tyrosine kinase inhibitor (TKI)), and immunotherapy, with platinum dual drug chemotherapy being the most common approach. However, most patients are unsuitable for chemotherapy due to severe adverse effects (33). One study reported that RFA significantly improved survival in patients with stage III to IV NSCLC, with a mean survival of 4.4 months in untreated patients and 13.6 months in those treated with RFA (34). Zhou et al. found that RFA offered pain alleviation to most advanced NSCLC patients with intractable pain who were resistant to radiation and chemotherapy. In this study, 23 of 40 patients continued to receive other therapies, such as chemotherapy, radiation, and TKI, after RFA. After four weeks of follow-up, 30% of patients had complete pain relief, 37.5% had partial pain relief, and 32.5% had no pain relief. Meanwhile, the pain was relieved significantly at the 24-hour, 72-hour, and 4-week follow-ups compared with baseline pain levels (35). This study suggests that palliative “debulking” of the tumor with RFA can greatly alleviate tumor pain. However, there are few other studies on the palliative treatment of RFA for pain relief in advanced NSCLC.

Several studies have described the synergistic effect of RFA in combination with chemotherapy in the treatment of advanced NSCLC, and the efficacy is better than that of any single therapy. Lee et al. reported that RFA combined with chemotherapy improved survival in patients with advanced NSCLC. They proposed a maximum tumor size of 3 to 5 cm for complete ablation of NSCLC (34). Li et al. also demonstrated the safety and efficacy of RFA as a supplementary therapy following systemic chemotherapy in patients with advanced NSCLC, with a median OS and PFS of 14 months and 16 weeks, respectively (36). Similarly, another study also reported that the combination of RFA and chemotherapy showed a significant effect on advanced NSCLC. The RFA with chemotherapy group had a much greater efficacy rate and a significantly lower progression rate than the RFA or chemotherapy alone group (37). Overall, the number of studies combining RFA with chemotherapy to treat advanced NSCLC is gradually increasing. This strategy is anticipated to establish a new paradigm for the treatment of advanced NSCLC, as it can improve local tumor control and prolong patient survival without significantly increasing adverse effects.

### RFA in metastatic lung cancer

Local ablation for metastatic lung cancer aims to prolong survival and achieve adequate tumor control. Many published studies have proven the efficacy of RFA in the treatment of metastatic lung cancer, particularly oligometastatic diseases, i.e., tumors of a limited size and number (generally considered to be <5 metastases at ≤ 3 sites) (38). The largest trial to evaluate the efficacy of RFA in the treatment of metastatic lung cancer was undertaken by T. de Baère et al. In this study, 566 patients with primary tumors in the colon, rectum, kidney, and soft tissue were treated with RFA. The median OS for the entire cohort was 62 months; the 1-, 2-, 3-, 4-, and 5-year OS rates were 92.4%, 79.4%, 67.7%, 58.9%, and 51.5%, respectively; the 1-, 2-, 3-, and 4-year PFS rates were 40.2%, 23.3%, 16.4%, and 13.1%, respectively; and the 1-, 2-, 3-, and 4-year local tumor progression rates were 5.9%, 8.5%, 10.2%, and 11.0%, respectively. In a multivariate analysis, the location of the primary tumor, absence of a disease interval, tumor size > 2 cm, and the occurrence of more than three metastases were all associated with OS (24). In addition, multiple studies on RFA in patients with lung metastases from colorectal cancer have shown that RFA significantly improves the long-term survival and median OS of such patients (17, 25–27). The first prospective study reported that the 1- and 2-year OS rates were 89% and 66%, respectively, and the 1- and 2-year tumor-specific survival rates were 91% and 68%, respectively (17). A retrospective study reported comparable outcomes, with OS rates of 95.2%, 65.0%, and 51.6% at 1, 3, and 5 years, respectively, and a median OS of 67 months for patients with lung metastases from colorectal cancer treated with RFA (25). With the advancement of ablation technology and patient selection, OS has increased gradually. Zhong et al. reported a median OS of 52 months, with OS rates of 96.7%, 74.7%, 44.1%, 27.5 and 16.3% at 1, 3, 5, 7 and 9 years, respectively, and PFS rates of 66.7%, 31.2%, 25.9%, 21.2% and 5.9%, respectively (27). Hiyoshi et al. found that the presence of extrapulmonary metastases and maximum tumor size > 15 mm were independent prognostic factors for PFS and OS in patients with lung metastases from colorectal cancer treated with RFA (26).

Combination therapy is also widely used in metastatic lung cancer. Chua et al. reported the results of RFA combined with systemic chemotherapy in 100 patients with lung metastases from colorectal cancer, with a median OS of 36 months and 1-, 2-, 3-, and 5-year OS rates of 87%, 66%, 50%, and 30%, respectively, and a reduced postoperative complication rate, indicating that the combination of chemotherapy and RFA offers the potential for disease control and improved survival in patients with colorectal lung metastases (28). Sano et al. considered a combination of RFA and surgical resection to be a viable treatment option for patients with metastatic lung cancer to increase cure rates and avoid highly invasive surgery (39). Another study demonstrated that RFA combined with systemic therapy is a safe and effective treatment strategy for patients with lung metastases of renal cell carcinoma, with great OS and prolonged systemic treatment-free survival (40). Hasegawa et al. treated 17 patients with multiple metastatic lung cancers using a combination of surgery and RFA. By surgically removing external or large tumors and performing RFA on internal or small tumors, all lung metastases were treated while maintaining lung function, and there was no local recurrence during the follow-up. The 1- and 5-year OS rates were 100% and 88%, respectively, and the disease-free survival rates were 48% and 32%, respectively, demonstrating that combining surgery and RFA provides favorable outcomes for lung metastases (29). In addition to the previously mentioned therapeutic modalities, RFA combined with immunotherapy has great prospects. Growing clinical evidence suggests that RFA induces coagulative necrosis of tumor cells and leads to the *in situ* release of large amounts of cellular debris, which can act as a source of tumor antigens and trigger an antitumor immune response in the host but cannot eradicate the tumor cells. The combination of RFA with immunotherapy is required to boost RFA-induced immune responses to achieve systemic and durable antitumor immunity, prevent recurrence, and improve PFS in cancer patients (41–43).

## Microwave ablation (MWA)

**Figure 3:**
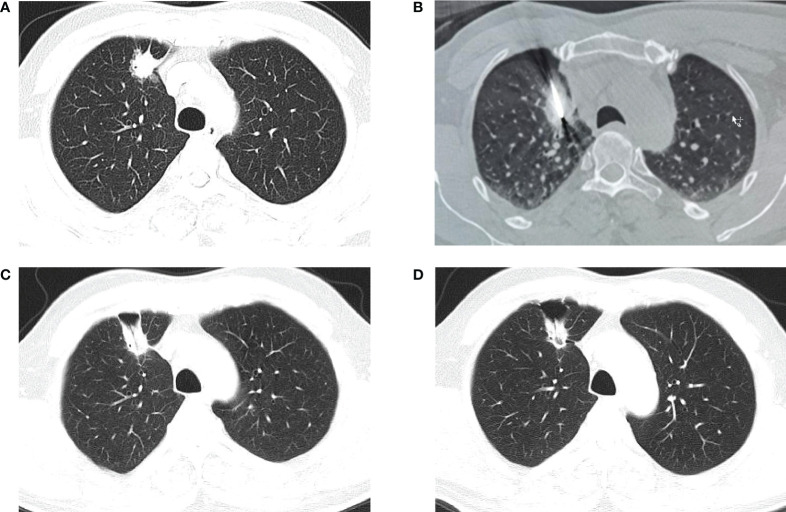
CT-guided percutaneous MWA. **(A)** CT scan performed before MWA, showing a lesion in the upper right lobe. **(B)** CT−guided percutaneous MWA of the lesion. **(C)** CT scan performed 1 month after the procedure. **(D)** CT scan performed 4 months after the procedure.

MWA is a rapidly developing local treatment for lung tumors as a local thermal ablation technique. Its efficacy and safety were first demonstrated in a large study of 50 lung cancer patients by Wolf et al. in 2008 (44). Subsequent studies have shown that MWA can provide excellent local control, safety, and survival rates for both primary and metastatic lung cancer. The principle is that through a needle inserted into the tumor, electromagnetic microwaves (915 or 2450 MHz) are transmitted directly to the tumor tissue. The polar molecules in the tumor tissue vibrate rapidly in the microwave electromagnetic field, causing collisions and mutual friction between molecules and generating heat, which rapidly raises the tissue temperature to 60-150°C, resulting in coagulative necrosis of tumor cells (45). Unlike RFA, MWA does not require current and skin grounding pads and allows the use of multiple probes. MWA can also achieve shorter ablation times, higher intratumor temperatures, larger ablation areas, and fewer heat dissipation effects. These characteristics make it more appropriate for complete ablation in patients with large tumors or tumors close to larger vessels or airways (46–48). The MWA process is displayed in **Figure 3**. Several studies of MWA in lung tumors are summarized in **Table 2**.

**Table 2:** Summary of published studies of MWA in lung tumors.

Author, Year	Type	Therapy	Tumor	No. of Patients	Median/mean follow- up (mo)	Mean size (cm)	Median OS (mo)	OS (%)			
								1- year	2-year	3-year	5- year
Yang et al., 2014 (49)	R	MWA	Stage I NSCLC	47	30.0	–	33.8	89.0	–	43.0	16.0
Yao et al., 2018 (50)	R	MWA	Stage I NSCLC	54	–	3.0	71.6	100.0	–	92.6	50.0
Han et al., 2019 (51)	R	MWA	Stage I NSCLC	63	21.0	2.7	50.0	97.1	92.6	63.4	32.6
Pusceddu et al., 2019 (52)	R	MWA	Advanced NSCLC	53	28.1	5.0	21.5	78.2	48.3	34.8	18.3
Zhong et al., 2019 (53)	R	MWA	Advanced NSCLC	78	18.0	–	–	93.6	87.7	71.7	–
Wei et al., 2020 (54)	P	MWA & chemotherapy	Stage IV NSCLC	148	13.1	3.6	–	–	–	–	–
Wei et al., 2015 (55)	R	MWA & chemotherapy	Advanced NSCLC	39	11.2	3.8	21.3	–	–	–	–
Xu et al., 2022 (56)	R	MWA & DEB-BACE	ASTRI-NSCLC	28	21.7	6.2	8.0	28.6	–	–	–
Vogl et al., 2011 (57)	P	MWA	Metastatic lung tumors	80	–	–	–	91.3	75.0	–	–
Meng et al., 2021 (58)	R	MWA	Metastatic lung tumors	32	32.0	–	36.0	96.9	75.0	53.3	17.8
Iezzi et al., 2021 (59)	P	MWA	Primary and secondary lung tumors	54	–	2.2	–	98.0	71.3	–	–
Liu et al., 2019 (60)	–	MWA & percutaneous coaxial biopsy	Primary and secondary lung tumors	23	31.0	1.3	–	91.3	69.6	60.9	–

MWA, microwave ablation; NSCLC, non-small cell lung cancer; DEB-BACE, drug-eluting bead bronchial arterial chemoembolization; ASTRI-NSCLC, advanced and standard treatment-refractory/ineligible NSCLC; mo, months; OS, overall survival; P, prospective; R, retrospective.

### MWA in early-stage lung cancer

Although not as extensively researched as RFA, MWA is becoming increasingly popular for image-guided percutaneous lung ablation. A growing number of clinical studies have shown that MWA is an effective treatment for early-stage NSCLC. Yang et al. reported a median OS of 33.8 months after MWA among 47 patients with stage I NSCLC. The OS rates at 1, 3, and 5 years were 89%, 43%, and 16%, respectively, and the local control rates at 1, 3, and 5 years were 96%, 64%, and 48%, respectively. Moreover, they discovered improved survival rates for tumors ≤ 3.5 cm in diameter (49). In a subsequent report on MWA for 104 patients with stage I NSCLC, 23.1% of patients had local recurrence and were treated with MWA again. At 1, 2, 3 and 5 years, the OS of patients without local recurrence was 100%, 74.6%, 60.6%, and 27%, respectively, whereas the OS of patients with repeated MWA was 96.4%, 69.5%, 60.6%, and 26.1%, respectively, indicating that repeat MWA is a safe and effective treatment for local recurrence with no adverse effects on survival (61). Yao et al. found that for stage I NSCLC, MWA yielded similar outcomes to lobectomy, with 1-, 3-, and 5-year OS rates of 100%, 92.6, and 50% for MWA and 100%, 90.7%, and 46.3% for lobectomy, respectively (50). The possibility that early NSCLC patients would not receive any treatment grows with age, and their survival is not optimistic. MWA has also been shown to be safe and effective in early-stage NSCLC patients aged 80 years and older, with OS rates of 97.1%, 92.6%, 63.4%, 54.4%, and 32.6% at 1, 2, 3, 4, and 5 years and cancer-specific survival rates of 97.9%, 97.9% and 69.4% at 1, 2 and 3 years, respectively. This study supports the use of MWA to treat elderly patients, improving their prognosis (51).

### MWA in advanced lung cancer

Although there is evidence that MWA is a promising therapeutic option for advanced lung cancer, there are few long-term follow-up data. In 53 patients with advanced NSCLC, Pusceddu et al. reported OS rates of 78.2%, 48.3%, 34.8%, and 18.3% at 1, 2, 3, and 5 years after MWA, respectively, and that OS was considerably lower in patients with tumors ≥4 cm than in those with smaller tumors (52). Zhong et al. explored the impact of MWA on patients with NSCLC and found a local progression rate of 20.5% in patients with advanced disease, with 81.3% of local progression occurring in tumors > 3 cm in diameter (53). MWA combined with chemotherapy can improve PFS and OS in patients with advanced NSCLC (3, 54, 55, 62). Wei et al. conducted a prospective multicenter randomized controlled trial that revealed a median PFS of 10.3 months in the combined group compared with 4.9 months in the chemotherapy group (54). Two recent studies have also indicated that MWA in combination with chemotherapy for advanced NSCLC is superior to chemotherapy alone in terms of efficacy, disease control, and prolonged patient survival and could be promoted in clinical research (63, 64). It has also been shown that MWA combined with radiotherapy is superior to radiotherapy alone in the treatment of advanced NSCLC (65, 66). In addition, Xu et al. compared the outcomes of drug-eluting bead bronchial arterial chemoembolization (DEB-BACE) alone and combined MWA for the treatment of advanced and standard treatment-refractory/ineligible NSCLC (ASTRI-NSCLC) and found that the 6-month OS of the combination and noncombination groups was 78.6% and 59.2%, respectively, the 6-month PFS was 75% and 22.4%, respectively, and the overall disease control rate was significantly higher in the combination group, indicating that for ASTRI-NSCLC, MWA combined with DEB-BACE is superior to DEB-BACE alone (56). In conclusion, MWA is safe, effective, and worthy of promotion in the treatment of patients with advanced NSCLC, and there is much potential for future research.

### MWA in metastatic lung cancer

MWA has also been shown to be safe and effective in the treatment of metastatic lung cancer. One of the earliest published prospective studies reported 1- and 2-year OS rates of 91.3% and 75%, respectively, and a local recurrence rate of 26% in 80 patients with metastatic lung cancer treated with MWA. Treatment success was significantly associated with tumor size < 3 cm and peripheral lesions (57). Meng et al. effectively treated lung metastases from breast cancer with MWA. The median OS was 36 months, and the OS rates at 1, 2, 3 and 5 years were 96.9%, 75%, 53.3%, and 17.8%, respectively (58). Iezzi et al. performed MWA on 54 patients with primary and metastatic lung cancer and reported an OS of 98.0% and 71.3% at 1 and 2 years, respectively, and an local tumor progression rate of 24.7%, indicating that MWA is a repeatable, safe, and effective treatment for malignant lung cancer (59). Liu et al. found that CT-guided percutaneous coaxial biopsy combined with MWA for metastatic lung cancer reduced biopsy complications and improved patients’ quality of life, prolonged survival, and increased survival rates (60). In conclusion, MWA is as effective and safe as RFA for the ablation of metastatic lung cancer. Identifying whether patients with metastatic lung cancer should undergo ablation, the timing of ablation, and the lesion selection is complex and requires a multidisciplinary assessment.

## Cryoablation (CA)

Argon-helium cryoablation is the most established cryoablation technique used in clinical practice. This technique is based on the Joule-Thompson theory. It employs helium and argon as heat and cold media, with high-pressure argon freezing the tumor tissue to -140°C and helium rapidly heating it from -140°C to 40°C. This continuous freeze−thaw process kills and destroys tumor cells by causing protein denaturation, cell dehydration, membrane rupture, and microvascular thrombosis (67). Hinshaw et al. suggested using three freezing cycles, which not only creates a larger ablation area but also shortens the ablation time (68). In 2005, Wang et al. reported the clinical application of CA in treating lung cancers for the first time. They performed CA on 234 tumors and achieved 98.7% and 87.2% complete tumor hockey coverage for peripheral tumors < 4 cm and > 4 cm, respectively (69).

**Figure 4:**
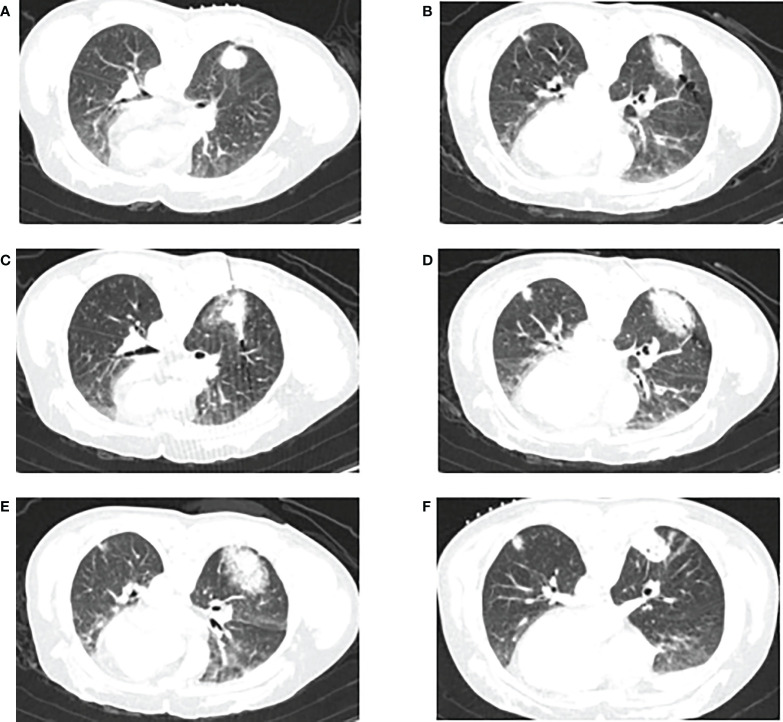
CT-guided percutaneous CA. **(A)** CT scan performed before CA, showing a lesion in the lower right lobe. **(B–E)** CT−guided percutaneous CA of the lesion. **(F)** CT scan performed 1 month after the procedure.

One of the advantages of CA over other thermal ablations is that it can evaluate the ablation site during surgery, enabling real-time treatment optimization (8). In addition, since the analgesic effect of freezing reduces both perioperative and postoperative patient pain, ablation can be performed under conscious sedation or local anesthesia, even if the target lesion is adjacent to the chest wall. CA does not disrupt the collagenous structure of the target tissue, making the treatment of tumors near the hilum or central airway safer (70–72). The CA process is displayed in **Figure 4**. Several studies of CA in lung tumors are summarized in **Table 3**.

**Table 3:** Summary of published studies of CA in lung tumors.

Author, Year	Type	Therapy	Tumor	No. of Patients	Median/mean follow- up (mo)	Mean size (cm)	Median OS (mo)	OS (%)			
								1- year	2-year	3-year	5- year
Yamauchi et al., 2012 (73)	R	CA	Stage I NSCLC	22	23.0	1.4	68.0	–	88.0	88.0	–
Moore et al., 2015 (74)	R	CA	Stage I NSCLC	45	51.0	–	–	89.4	–	78.1	67.8
McDevitt et al., 2016 (70)	R	CA	Stage I NSCLC	25	11.1	–	43.0	100.0	–	63.0	–
Gu et al., 2011 (75)	–	CA & molecular target therapy	Advanced NSCLC	18	–	–	–	66.7	–	–	–
Yamauchi et al., 2016 (76)	R	CA	Metastatic lung tumors	24	40	1.3	43.0	91.0	–	59.6	–
de Baere et al., 2015 (77)	P	CA	Metastatic lung tumors	40	–	1.4	–	97.5	–	–	–
Callstrom et al., 2020 (78)	P	CA	Metastatic lung tumors	128	–	1.0	–	97.6	86.6	–	–
de Baère T et al., 2021 (79)	P	CA	Metastatic lung tumors	40	–	1.4	–	97.5	84.3	63.2	46.7

CA, cryoablation; NSCLC, non-small cell lung cancer; TCM, traditional Chinese medicine; mo, months; OS, overall survival; P, prospective; R, retrospective.

### CA in early-stage lung cancer

CA is a relatively safe therapeutic option for patients with inoperable early-stage lung cancer, and the goal of treatment should be to achieve a radical cure. However, unlike RFA and MWA, experience with CA for early-stage lung cancer is limited. Yamauchi et al. reported the first results of CA specifically for inoperable stage I NSCLC patients, with a total of 25 treatments in 22 patients. They found a local control rate of 97%, a median OS of 68 months, and a 3-year OS of 88% (73). Another early study retrospectively evaluated the 5-year OS and PFS of 45 patients with stage I NSCLC treated with CA, which were 67.8% and 87.9%, respectively, and the local recurrence rate was 36.2% (74). McDevitt et al. reported 1- and 3-year OS rates of 100% and 63%, respectively, in 25 patients with stage I NSCLC treated with CA. Tumors with a maximum diameter > 3 cm were associated with an increased risk of local progression (70). Nomori et al. used liquid nitrogen CA to treat patients with T1N0M0 NSCLC and discovered that local recurrence occurred in 10 of 101 patients, with no recurrence in the tumor size group below 1.2 cm (0%), one recurrence in the 1.3-1.7-cm group (4%), and nine recurrences in the 1.8-cm and above group (33%), indicating that smaller tumors were associated with better local control (80). Given these findings and the minimally invasive nature of the technique, CA is a viable alternative for patients with unresectable early-stage lung cancer. As with any form of thermal ablation, tumor size is a significant risk factor for local progression.

### CA in advanced lung cancer

Available studies have shown that CA can be used to treat advanced lung cancer that cannot be surgically resected with reasonable local tumor control (81). Niu et al. compared the therapeutic effects of CA and palliative treatment in patients with stage IV lung cancer. After 6.5 years of follow-up, they found that OS in the CA group was considerably longer than that in the palliative treatment group. They also found that multiple repeated CA treatments may be superior to single therapy (82). Gao et al. reported that CA contributes to effective local tumor therapy in patients with stage IIIB/IV NSCLC following the failure of radiotherapy, with a one-year OS of 81.8% and a PFS of 27.8% (83).

A potential advantage of CA is that the cellular contents of damaged cancer cells are not affected and are delivered to immune cells upon cell rupture, prompting an antitumor immune response that may enhance the efficiency of later immunotherapy (72, 84). Accordingly, several studies have investigated the efficacy of CA in combination with immunotherapy for the treatment of patients with advanced lung cancer (85, 86). In a study by Yuanying et al., 161 patients with stage IV NSCLC were treated with a multimodal regimen consisting of platinum-based chemotherapy, intravenous dendritic cell cytokine-induced killer (DC-CIK) immunotherapy, and CA. Patients receiving CA in combination with chemotherapy or immunotherapy had a longer median OS than those receiving chemotherapy or immunotherapy alone (18 and 17 months vs. 8.5 and 12 months). Those receiving CA in combination with immunotherapy and chemotherapy had a median survival of 27 months, indicating that the combination of the three therapies was the optimal treatment for this group of patients (86). Lin et al. prospectively reported a synergistic effect of CA in combination with allogeneic natural killer (NK) cell immunotherapy for advanced NSCLC, which not only improved patients’ immune function and quality of life but also significantly enhanced remission rates and disease control rates (85). These studies indicate the efficacy of combining CA with immunotherapies such as DC-CIK or NK-cell therapy. Currently, CA in combination with immune checkpoint inhibitors (ICIs) has shown encouraging results in breast and prostate cancer, but the efficacy in advanced NSCLC is unclear; thus, further studies are needed to characterize the therapeutic effects of CA combined with ICIs in lung cancer (87).

CA may also have a significant long-term effect when combined with other therapies. A randomized controlled trial showed that the combination of CA and molecularly targeted therapy significantly improved 1-year survival and rates of disease stabilization and progression in patients with advanced NSCLC (75). In China, green cancer therapy is a new form of treatment that combines CA with traditional Chinese medicine (TCM). Sun et al. reported that this approach improved survival time and quality of life for patients with stage IIIb/IV NSCLC compared with chemotherapy alone, providing a novel treatment strategy for patients with advanced cancer (88). A previous study also reported the efficacy of CA combined with TCM in treating elderly individuals or those with advanced lung cancer (89). Overall, CA is a safe and effective ablative therapy for patients with advanced lung cancer. However, all possible complications in the treatment should be prevented before, during, and after the operation.

### CA in metastatic lung cancer

Despite the limited survival data, CA has also been shown to be effective in the treatment of metastatic lung cancer (70, 76). Yamauchi et al. reported a median OS of 43 months in 24 patients with pulmonary metastases from colorectal cancer who were treated with CA. The 1- and 3-year OS rates were 91% and 59.6%, respectively, and the 1- and 3-year local PFS rates were 90.8% and 59%, respectively (76). de Baere et al. published the first prospective multicenter study of CA for metastatic lung cancer, showing a one-year OS of 97.5% and local tumor control rates of 96.6% and 94.2% at 6 months and 1 year, respectively, demonstrating that CA is a safe and effective treatment for metastatic lung cancer (77). Two recent prospective multicenter trials, SOLSTICE and ECLIPSE, showed reasonable local control of CA for metastatic lung cancer (78, 79). In the SOLSTICE trial, 224 patients with lung metastases had an OS of 97.6% and 86.6% at 1 and 2 years and local tumor control rates of 85.1% and 77.2% after the first CA, respectively. At 1 and 2 years after re-CA for locally recurrent tumors, the local tumor control rates increased to 91.1% and 84.4%, respectively (78). In the ECLIPSE trial, the local tumor control rates at 3 and 5 years were 87.9% and 79.2%, respectively, and the OS rates at 3 and 5 years were 63.2% and 46.7%, respectively, in 60 cases of CA-treated lung metastases (79). Although these results are highly encouraging, further studies are needed, particularly for local tumor control and long-term follow-up of OS.

## Future prospects

Percutaneous ablation must be conducted through the pleura, resulting in a high incidence of pneumothorax and an increased risk of perivascular lesions, while ablation of peripheral malignancies through the natural lumen under the guidance of tracheoscopy can reduce the occurrence of such complications. Bronchoscopic ablation is an emerging technique for the treatment of lung tumors. Advances in electromagnetic navigation and robot guidance make it possible to reach central and peripheral tumors through the bronchus, allowing for high-dose local ablation. Currently, bronchoscopic ablation is often used to treat central airway lesions caused by malignant tumors, while the treatment of peripheral malignant lesions is still in the early stages of research, and it is unclear whether bronchoscopic ablation also has a relatively imprecise targeting. Therefore, more large-scale research and long-term follow-up of bronchoscopic tumor ablation are still needed in the future (12).

As a minimally invasive and accurate tumor treatment technology, thermal ablation usually produces a weak antitumor immune response and cannot eradicate all tumor cells. Therefore, the focus of the current research is whether it can enhance the synergistic effect through the combination of tumor immunotherapy. Several studies have demonstrated that thermal ablation combined with interleukin-2 (90), immunostimulant OK-432 (91), granulocyte-macrophage-colony-stimulating factor (92), toll-like receptor agonists (93), tumor necrosis factor (94), dendritic cells (95–97), dendritic cell-activated cytokine-induced killer cells (86), NK cells (85), and anti-CTLA-4 (98) can promote the body to produce a more powerful antitumor immune effect to obtain better efficacy. However, these studies are still in the preliminary stage, and further studies on the efficacy and safety of thermal ablation combined with immunotherapy are expected in the future.

During the ablation process, precise probe placement is crucial for achieving technical success and ensuring adequate ablation margins to avoid local tumor recurrence. Moreover, incorrect probe placement may result in severe complications, which can threaten patient safety (99, 100). Robot-assisted ablation, which refers to the use of software and navigation systems to assist in the planning of ablation procedures and the placement of probes, has achieved success in the treatment of tumors in recent years, resulting in a high degree of accuracy and reduced radiation dose, with the potential to improve tumor ablation outcomes (101–103). However, most published studies have focused on robotic system-assisted ablation of liver cancer. Its application in the ablation of lung tumors has not been discovered. Therefore, more lung cancer patient groups will be required in the future to investigate the application of robotic systems in lung tumor ablation.

## Conclusion

Minimally invasive treatment is one of the future directions in the treatment of lung malignancies. Image-guided percutaneous tumor ablation has shown safety and efficacy in treating primary and metastatic lung cancer, and it is an effective treatment strategy for inoperable patients. RFA, MWA, and CA are the most widely used lung ablation techniques. They are frequently combined with other treatments to improve local tumor control, achieve better outcomes, reduce complications, and improve patients’ quality of life. Currently, there is no conclusive evidence to determine the most appropriate ablation modality. Further large-scale data accumulation is needed in the future, particularly for long-term outcomes and comparisons with other therapies.

## Author contributions

YS carried out the primary literature search, drafted and revised the manuscript. GL, YW, YC, and CL helped modify the manuscript. BZ provided with available image data. WZ and JL revised and edited the final version of the manuscript. All authors contributed to the article and approved the submitted version.

## Funding

The study was supported by National Natural Science Foundation of China (82102157), Hunan Provincial Natural Science Foundation for Excellent Young Scholars (2022JJ20089), Hunan Provincial Natural Science Foundation of China (2021JJ40895), the science and technology innovation Program of Hunan Province (2020SK53423), the Research Project of Postgraduate Education and Teaching Reform of Central South University (2021JGB147, 2022JGB117) and the Clinical Research Center For Medical Imaging In Hunan Province (2020SK4001), the science and technology innovation program of Hunan Province (2021RC4016). Central South UniversityResearch Programme of AdvancedInterdisciplinary Studies (2023QYJC020).

## Conflict of interest

The authors declare that the research was conducted in the absence of any commercial or financial relationships that could be construed as a potential conflict of interest.

## Publisher’s note

All claims expressed in this article are solely those of the authors and do not necessarily represent those of their affiliated organizations, or those of the publisher, the editors and the reviewers. Any product that may be evaluated in this article, or claim that may be made by its manufacturer, is not guaranteed or endorsed by the publisher.
